# Electrospun Polymeric Film in Red BF-4B Dye Degradation

**DOI:** 10.3390/polym17192669

**Published:** 2025-10-02

**Authors:** Andressa Giombelli Rosenberger, Eduarda Ballmann, Fabiana da Silva Lima, Josiane Caetano, Douglas Cardoso Dragunski, Edvani Curti Muniz, Aparecido Nivaldo Módenes

**Affiliations:** 1 Engineering and Exact Sciences Center, Toledo Campus, State University of West Paraná, 645, Rua da Faculdade, Toledo 85903-000, PR, Brazil; duda_ballmann@hotmail.com (E.B.); fabii_lima@hotmail.com (F.d.S.L.); dcdragunski@gmail.com (D.C.D.); anmodenes@yahoo.com.br (A.N.M.); 2 Department of Chemistry, State University of Maringa (UEM), Maringá 87020-900, PR, Brazil; curtimuniz@gmail.com; 3 Department of Chemistry, Federal University of Piauí (UFPI), Teresina 64049-550, PI, Brazil

**Keywords:** electrospinning, PBAT/PLA/TiO_2_ fibers, photocatalytic degradation, Red BF-4B dye, environmental remediation

## Abstract

This work aims to apply polymeric PBAT/PLA fibers electrospun with TiO_2_ in the photodegradation of the dye Red BF-4B in an aqueous solution and the dye’s subsequent reuse. Initially, the influence of the solution pH was evaluated, and the results showed more significant dye degradation at pH values below the pHpcz (7.42). Kinetic studies show that at 15 mg·L^−1^, the highest percentage of degradation occurs at 600 min of reaction time; however, degradation equal to (or greater than) 65% was observed at all evaluated concentrations, with the kinetic data being well fitted by the pseudo-first-order model. Additional studies demonstrated the reuse of polymeric films for dye removal, with removal efficiencies ranging from 86.60% to 93.07% over six consecutive reuse cycles. Each cycle consisted of a 600 min removal process, simulating repeated practical applications. After the photocatalytic process, the polymeric fibers remained cylindrical, with several fractures. Diameter decreases of 31.61% and 7.95% were observed after the first and sixth cycles, respectively, with possible exposure of TiO_2_. The vibrational spectra indicate changes in the bands at 1755 and 1714 cm^−1^, attributed to C=O (PLA) and C-O (PBAT) stretches, respectively, suggesting a possible conformational change in the polymers. The thermal profiles showed only slight changes after the cycles. X-ray diffractograms indicate that degradation of the polymeric matrix leads to greater exposure of the embedded TiO_2_ particles. The combined results from different characterization techniques provide evidence of the degradation of the polymeric material.

## 1. Introduction

The environmental consequences resulting from global economic development have intensified air, soil, and water pollution. The growing consumer demand for a wide range of chemicals has mainly affected aquatic ecosystems. The textile and dye industry plays an essential economic role worldwide [1]. Billions of new garments are produced and sold globally each year, and prospects indicate that the market will continue to grow in the coming years [2,3].

Despite the practical economic benefits, the textile and dye industry contributes significantly to environmental pollution, especially during the dyeing and finishing stages [4,5]. This occurs because, in these stages, a significant portion of the dye does not adhere to the textile fibers and is released into the environment, causing several changes in the physical, chemical, and biological properties of aquatic ecosystems [6,7].

The class of azo dyes has been one of the most widely used by the textile industry due to the high-intensity colorings of these dyes [8,9]. However, azo dyes have complex molecular structures, are chemically stable, exhibit low biodegradability, and have high solubility in water, making them difficult to remove through conventional wastewater treatment systems. Additionally, they possess toxic and carcinogenic potential [8,10,11,12,13,14,15]. Therefore, it becomes necessary to employ tertiary and additional treatments to ensure their complete removal [16].

The main physical, chemical, and biological methods used for removing dyes from wastewater include chemical precipitation, ultrafiltration, aerobic and anaerobic microbial degradation, coagulation/flocculation, electrochemical treatments, reverse osmosis, adsorption, and advanced oxidation processes. Although many of these methods have been widely tested in laboratory settings and have demonstrated satisfactory efficacy, their industrial applications remain limited [17].

For instance, adsorption, despite being a simple and low-cost method, merely transfers pollutants from the effluent to a solid phase, generating secondary waste that requires additional treatment. Similarly, biological treatments are environmentally friendly but often fail to completely degrade the complex molecular structure of azo dyes. In addition, many of these conventional processes can be costly, require high energy consumption, and involve long operation times. Thus, the choice of an appropriate treatment is a decisive factor, governed by environmental and economic aspects such as associated costs and raw material requirements. Given these limitations, Advanced Oxidation Processes (AOPs) have emerged as a promising alternative, as they are capable of completely mineralizing dyes into non-toxic compounds [17].

Among the various technologies developed for textile wastewater treatment, heterogeneous photocatalytic processes have been extensively investigated and applied in recent years [18,19,20]. As the generation of a potent oxidizing agent, such as hydroxyl radicals, occurs at the catalyst’s surface, metallic semiconductors such as TiO_2_ are widely used due to their properties, chemical stability, non-toxicity, and efficiency in removing bacteria and organic contaminants [21,22]. However, the use of TiO_2_ becomes impractical due to the difficulty and high cost associated with recovering and separating these particles from the effluent [23]. With the aim of enhancing the separation and recyclability of metallic catalysts like TiO_2_, polymeric materials from renewable sources have become an interesting alternative. In this study, we utilize a blend of poly (lactic acid) (PLA) and poly (butylene adipate-co-terephthalate) (PBAT), taking advantage of the synergistic effects of both polymers [24,25].

PLA is a promising biopolymer, recognized for its bio-based origin, biodegradability under specific conditions, and biocompatibility. However, its properties are limited by low toughness, high brittleness, and a slow crystallization rate. To overcome these disadvantages, PLA is blended with PBAT, a flexible and ductile polyester. The addition of PBAT significantly improves the mechanical properties and processability of the final blend [26].

As a result, the blend becomes a more versatile and robust material, combining the sustainability of PLA with the enhanced mechanical performance of PBAT. This composite material can then be used as a durable and eco-friendly platform for the immobilization of catalysts, such as TiO_2_ [26].

Considering the environmental problem involving textile dyes, the immobilization of TiO_2_ nanoparticles in a biodegradable and compostable polymeric matrix of PBAT/PLA was used in this study. PBAT/PLA/TiO_2_ fibers were evaluated in heterogeneous photocatalytic processes involving the azo dye Red BF-4B, widely used in the textile industry. Morphological, structural, and thermal characterizations were also carried out after the photocatalytic processes, aiming to evaluate the viability and safety of this material during reuse.

## 2. Materials and Methods

### 2.1. Materials

The fibers were produced using the Ecovio^®^ F Blend C2224 polymer marketed by BASF (Ludwigshafen, Germany). This polymer consists of a biodegradable polymeric blend composed of 55% poly (butylene adipate-co-terephthalate) (PBAT), under the trade name Ecoflex^®^, and 45% poly (lactic acid) (PLA), derived from renewable sources. Due to its composition, the abbreviation PBAT/PLA was used to present the results of this work. The PBAT/PLA was solubilized in the solvents chloroform (CHCl_3_) and N,N-dimethylformamide (C_3_H_7_NO), supplied by Neon (Suzano, Brazil). The TiO_2_ nanoparticles (NPs) added to the polymeric solution were acquired from Sigma Aldrich (St. Louis, MO, USA) and consist of a mixture of rutile and anatase phases, in powder form, with particle size less than 100 nm and a purity level of 99.5%. The textile dye used for photocatalytic degradation was Reactive Red BF-4B, supplied by Textile Química Ltda. (São Paulo, SP, Brazil). This dye is cataloged in the Color Index as C.I. Reactive Red 195 (CAS 93050-79-4), and it has the molecular formula C_31_H_19_N_7_Na_5_O_19_S_6_Cl and a molar mass of 1136.32 g·mol^−1^.

### 2.2. Methods

#### 2.2.1. Preparation of Electrospun Fibers and Electrospinning Conditions

The fibers were produced through electrospinning, using polymeric solutions containing 15% (*w*/*v*) of PLA/PBAT, with the addition of TiO_2_ nanoparticles (10% *w*/*v*). The polymer was solubilized in a mixture of chloroform/N,N-dimethylformamide (DMF) solvents in a ratio of 85/15% (*v*/*v*), respectively [27,28]. For the complete solubilization of the PBAT/PLA polymer and suspension of the TiO_2_ NPs, the solution was kept under magnetic stirring for 48 h. Afterward, the PBAT/PLA/TiO_2_ solution was inserted into a Hamilton glass syringe (Ø = 0.70 mm). The parameters used for electrospinning were adjusted as follows: a flow rate of 1.80 mL·h^−1^ (SP100I Syringe Pump, WPI Inc., New York, NY, USA); a 16 cm distance between the needle tip and the metallic collector, where the fibers were deposited and pre-dried; an electric potential difference of 18 kV, applied using a high-voltage source (Bertan 30-R); temperature maintained at 25 °C; and ambient humidity controlled at approximately 50% [27,28].

#### 2.2.2. Heterogeneous Photocatalysis Experiments

After the PBAT/PLA/TiO_2_ electrospun fibers were obtained, they were used in photocatalytic studies to remove the Red BF-4B dye. For this, a discontinuous reactor with vertical irradiation was used, consisting of a beaker (49.73 mm × 122.04 mm, 250 mL capacity) coupled to a cooling system controlled by an ultra-thermostatic bath (Solab, Brazil), model SL-152. As a radiation source, a high-pressure xenon lamp, model E271ES Ultra-Vitalux (Osram, Brazil), was used, with a nominal power of 300 W. The light source and the dye solution were maintained separated by a distance of 28.0 cm, and the system’s temperature was kept at 21.0 °C. Under these conditions, UVA/UVB radiation was measured using a UM-200 radiometer (Precision Control Ltd., Selangor, Malaysia), with an intensity of 45 W·m^−2^. The polymeric films were held in the dye solution using a 3.0 cm diameter stainless steel ring. Each photocatalytic assay was conducted with 30 mL of dye solution. The evolution of the photocatalytic process was monitored by UV-Vis absorbance using a Shimadzu UV-1800 UV-Vis spectrometer (Kyoto, Japan) in the 200–800 nm spectral range, at predefined wavelength intervals.

Initially, the influence of pH on dye degradation was investigated. The experiments were carried out in batches for 600 min at pH 4.0, 6.0, 8.0, and 10.0. In each run, new circular polymeric films (4 cm diameter) of PBAT/PLA/TiO_2_ electrospun fibers were used. The film was immersed in 30 mL of Red BF-4B dye solution at a concentration of 20 mg·L^−1^. The tests were conducted in duplicate, and absorbance measurements were taken at 60 min intervals. Additionally, the pHpcz study of PBAT/PLA/TiO_2_ electrospun fibers was conducted according to the methodology described by Mimura [29]

After the pH analysis, batch photodegradation kinetics studies were carried out, with contact time evaluated from 0 to 600 min at concentrations of 15, 30, 45, and 60 mg·L^−1^. The tests were performed in duplicate. To obtain the kinetic data, 2.0 mL aliquots were removed from the liquid phase at predetermined intervals. After absorbance measurements, the aliquots were returned to the reaction medium. The reaction rate was determined by applying the zero-order, pseudo-first-order, and pseudo-second-order models. The models’ fits were evaluated considering the coefficient of linear determination (R^2^) and the root mean square error (RMSE).

In another set of tests, experiments were carried out using the polymeric electrospun fibers (PBAT/PLA/TiO_2_) over six successive degradation cycles to verify their reuse potential. In each cycle, the process was evaluated for 600 min in a Red BF-4B dye solution (30 mg·L^−1^), with the pH adjusted to 6.0.

#### 2.2.3. Characterization of the PBAT/PLA/TiO_2_ Polymeric Membrane After the Photocatalytic Process

After the photocatalytic studies, the polymeric membranes were characterized to verify possible changes in their structure, morphology, and composition.

To assess the fibers’ morphological structure and surface composition before and after the 1st and 6th cycles of the photocatalytic process, the samples were analyzed using scanning electron microscopy (SEM) coupled with energy-dispersive X-ray spectroscopy (EDS). For this, a scanning electron microscope (Tescan^®^, Warrendale, PA, USA), model Vega 3, with magnifications of 2000×, 10,000×, and 20,000×, coupled to an energy-dispersive X-ray detector (Oxford Instruments, Abingdon, UK), was used. The samples were fixed with double-sided carbon adhesive tape on the stubs and then coated with a gold layer approximately 5 nm thick using a metallizer (Denton Vacuum, Moorestown, NJ, USA), model Desk V.

SEM images with a magnification of 10,000× were used to measure the diameter of the fibers. Subsequently, 20 fibers (n = 20) were randomly selected from the given sample image, processed with the aid of ImageJ 2 software, and presented with their average diameters and respective standard deviations.

Fourier Transform Infrared Spectroscopy (FTIR) analysis was performed using a spectrophotometer (Perkin Elmer, model STA 6000, Waltham, MA, USA) equipped with an Attenuated Total Reflectance (ATR) module featuring a zinc selenide crystal, to evaluate the chemical structure of the PBAT/PLA/TiO_2_ electrospun fibers. The analyses were conducted at room temperature, with a scanning range between 4000 and 600 cm^−1^, and eight accumulations were acquired to obtain a resolution of 4 cm^−1^.

The thermal stability of PBAT/PLA/TiO_2_ electrospun fibers was evaluated by thermogravimetric analysis (TG-DTGA) using a thermal analyzer (Perkin-Elmer, Waltham, MA, USA), model STA 6000, equipped with a ceramic cell. Sample masses ranging from 5.0 to 10.0 mg were used, and the analyses were carried out under a dynamic nitrogen atmosphere (50 mL min^−1^), with a heating rate of 10 °C min^−1^, over a temperature range from 25 to 900 °C.

The degradation, melting, and crystallization properties of polymeric films were analyzed by differential scanning calorimetry (DSC) using a calorimeter (Shimadzu, Japan), model DSC-60. In each analysis, samples weighing between 5.0 and 10.0 mg were placed in sealed aluminum crucibles. The samples were heated from 25 to 190 °C at a heating rate of 10 °C min^−1^ under a dynamic nitrogen atmosphere (50 mL min^−1^).

The degree of crystallinity of the samples was determined by X-ray diffraction (XRD) using a diffractometer (Bruker^®^, Billerica, MA, USA), with a 2θ diffraction angle ranging from 0 to 120°, at 0.01° increments, using CuKα radiation (λ = 1.54184 Å) and a graphite monochromator.

**Figure 1:**
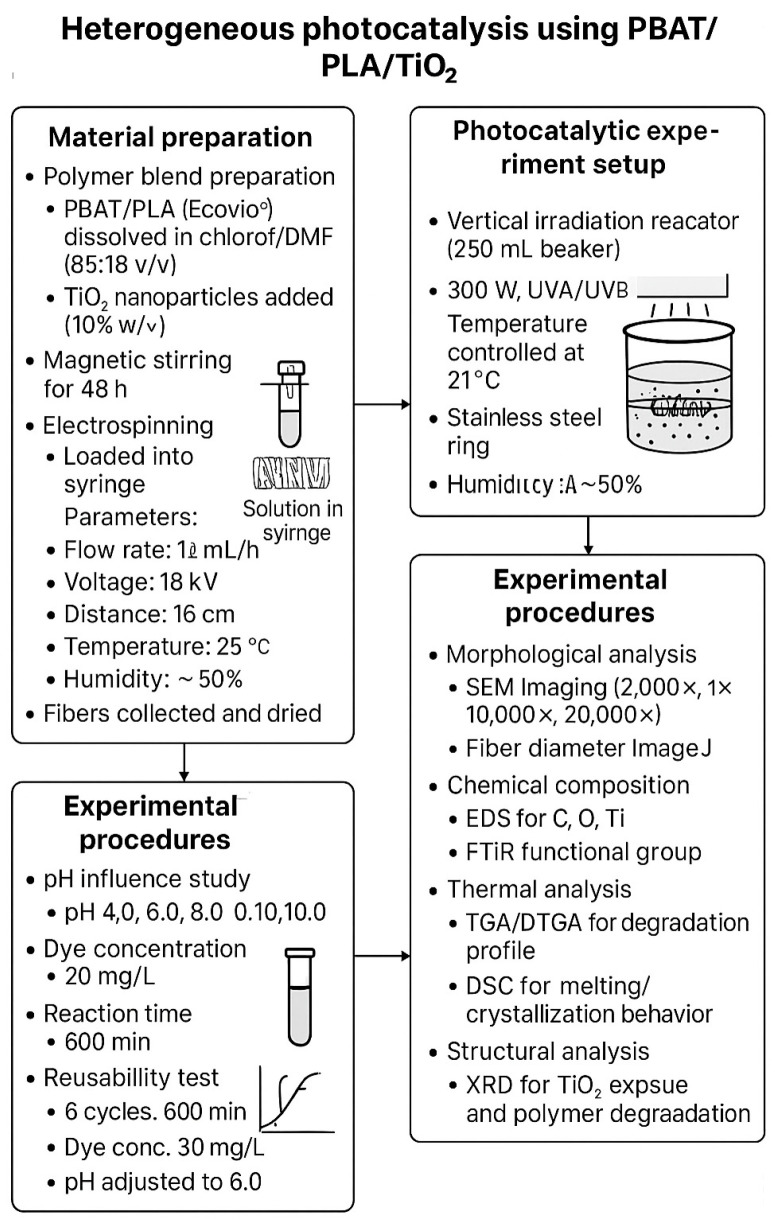
Schematic representation of the heterogeneous photocatalysis process using PBAT/PLA/TiO_2_ electrospun fibers for the degradation of Red BF-4B dye. The diagram illustrates the complete workflow, including polymer blend preparation, electrospinning, photocatalytic reactor setup, experimental procedures (pH influence, kinetic studies, and reuse cycles), and post-treatment characterization techniques (SEM, EDS, FTIR, TGA, DSC, and XRD).

To provide a comprehensive overview of the experimental methodology, a process flow diagram has been included (Figure 1). This diagram outlines the entire workflow of the heterogeneous photocatalysis experiments, beginning with the preparation of PBAT/PLA/TiO_2_ electrospun fibers, progressing through the photocatalytic reactor setup and experimental procedures—including pH influence, kinetic studies, and reuse cycles—and concluding with post-treatment characterization techniques such as SEM, EDS, FTIR, TGA, DSC, and XRD analyses. This visual representation aims to enhance the reader’s understanding of the experimental design and sequence.

## 3. Results and Discussion

**Figure 2:**
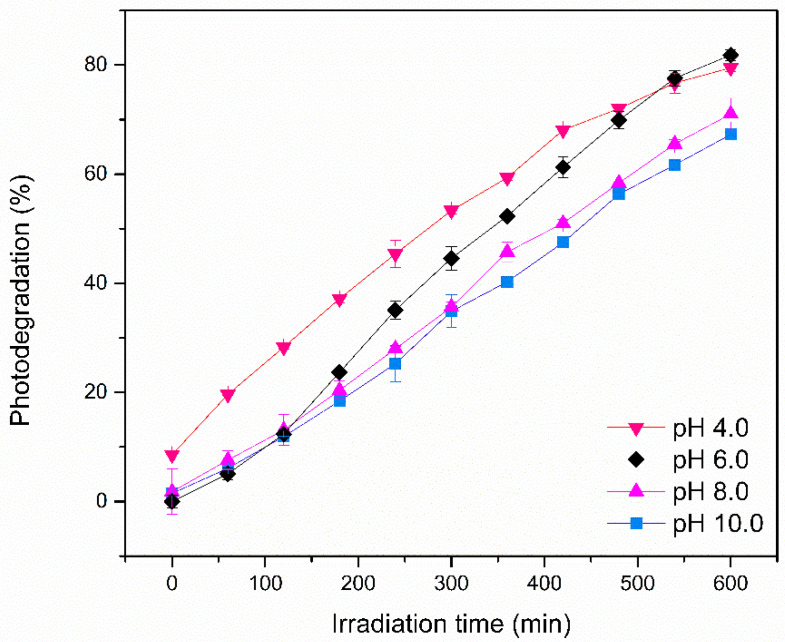
Degradation of Red 4B-BF dye using PBAT/PLA/TiO_2_ electrospun fibers at pH 4.0. 6.0. 8.0, and 10.0.

The influence of pH on the photodegradation of Red BF-4B dye using PBAT/PLA/TiO_2_ electrospun fibers was analyzed (Figure 2).

The results of heterogeneous photocatalysis with PBAT/PLA/TiO_2_ electrospun fibers at different pH levels (Figure 2) show that higher efficiencies were obtained at pH 4.0 and 6.0, with degradation yields (or percentage of dye removal) after 600 min of reaction time reaching 79.5% and 81.8%, respectively. However, at 420 min and pH 4.0, 68.08% of the dye had already been degraded. After 600 min, the removal rates at pH 8.0 and 10.0 were 71% and 67%, respectively, indicating a decrease in the photodegradation potential of PBAT/PLA/TiO_2_ electrospun fibers in basic media. This result is consistent with the pHpcz analysis, which suggested that at pH values below the pHpcz of PBAT/PLA/TiO_2_ fibers (7.42), the photocatalysis of the anionic Red BF-4B dye is favored due to the protonation of the polymeric fiber surface. Some studies report that TiO_2_ exhibits greater oxidizing activity at low pH; however, excess H^+^ ions can reduce the reaction rate [30]. The influence of pH is crucial, as reported by Sakarkar [31], since the surface charge of the polymeric film significantly affects the efficiency of pollutant removal. Table 1 presents the pH values measured before and after 600 min of photocatalytic reaction.

A decrease in all the tested pH values was observed. This can be attributed to the degradation of the PLA component of the polymeric film during the photocatalytic process. The breakdown of PLA’s ester bonds via hydrolysis leads to the formation of carboxylic acid end-groups and oligomers, which progressively lower the pH of the solution [32,33]. Due to this inherent characteristic of this system, it was decided to work at pH 6.0. In addition to greater efficiency, it is suggested that a slight adjustment in acidity may be necessary for industrial applications operating at a pH lower than 4.0. Brazilian law states that effluents must have a pH between 5.0 and 9.0 to be released into water bodies [34,35].

**Figure 3:**
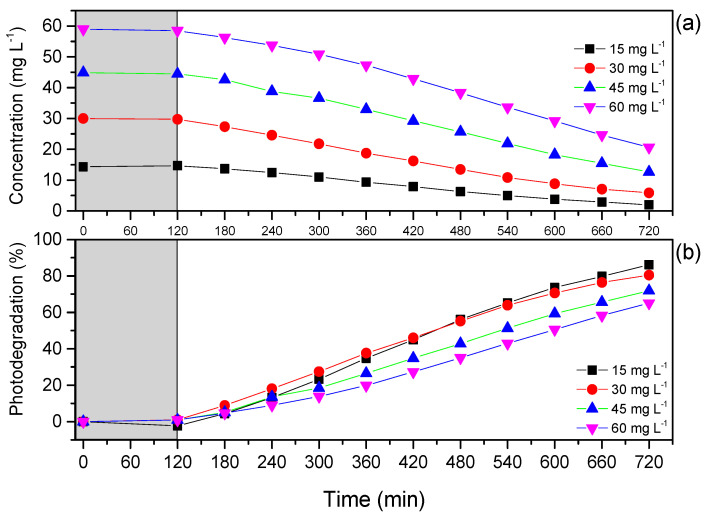
Degradation kinetics of Red 4B-BF dye at concentrations of 15 (■), 30 (●), 45 (▲), and 60 (▼) mg L^−1^: (**a**) concentration (mg L^−1^) versus time (min); (**b**) % of catalytic degradation versus time (min).

After establishing the working pH at 6.0, the photocatalytic degradation kinetics of the Red BF-4B dye catalyzed by PBAT/PLA/TiO_2_ electrospun fibers were studied using four different concentrations: 15, 30, 45, and 60 mg L^−1^ (Figure 3).

Due to the hydrophobic character of the PBAT/PLA/TiO_2_ electrospun fibers [36,37,38,39], we decided to insert the polymeric films into the dye solution for 120 min in the absence of light before the kinetic test started. In this case, as there is no resistance barrier, water penetrates the hydrophobic surface of the fibers through physical mechanisms such as encrustation and moistening [40].

It can be observed in Figure 3b that at the end of the reactions (600 min), the curve for 15 mg L^−1^ presented the highest percentage of degradation in comparison to the solutions of 30, 45, and 60 mg L^−1^. However, in all evaluated concentrations, the degradations reached values equal to or greater than 65%.

The increase in degradation observed at lower concentrations may be explained by the more significant interaction of ●OH radicals on the surface of the TiO_2_ photocatalyst immobilized on the electrospun fibers with the Red BF-4B dye molecules [41]. Also, lower concentrations allow more light to pass through the polymeric film [41], contributing to the efficiency of photodegradation.

The degradation rate depends on the likelihood of ●OH radicals forming on the catalyst surface and reacting with dye molecules. Several factors can influence these processes [41]. Similar results were observed by Saggioro [42], who used TiO_2_ P25 Degussa as a catalyst, and the photodegradation was performed in aqueous solutions of C.I. Reactive Black and C.I. Reactive Red 239 under artificial irradiation from a mercury lamp. The authors attributed the decrease in photodegradation efficiency to the reduction of hydroxyl radicals, because as the initial concentration of the dye increased, more dye molecules were adsorbed on the catalyst surface, occupying the active sites [42].

However, as shown in Figure 3a,b, it was observed that the PBAT/PLA/TiO_2_ electrospun fibers do not adsorb the dye molecules; thus, the most significant effect seems to be due to the protection against ultraviolet light that the high concentrations of dye provided. As the dye concentration in the solution increases, it starts to coat the surface of the photocatalyst [41,43]. Thus, it is understood that photons of light reach the catalyst surface more efficiently when the dye concentration is lower, promoting the formation of gap–electron pairs and the generation of hydroxyl radicals. Davis [44] explain that within the reactor, the incoming ultraviolet photons can be absorbed by both the photocatalyst and the dye in solution; thus, at higher dye concentrations, the path length of the photons entering the solution is reduced. Therefore, with the increase in the amount of dye, the number of free radicals that attack the dye molecules decreases [41].

**Figure 4:**
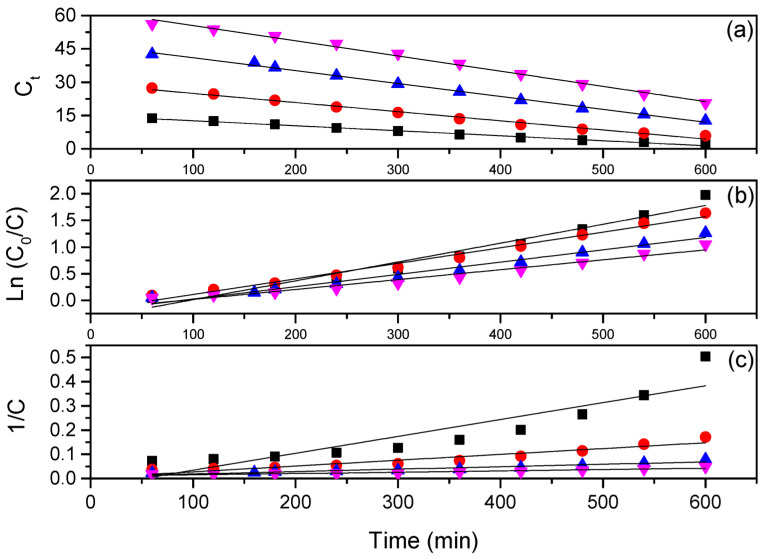
Kinetic profiles of zero-order (**a**), pseudo-first-order (**b**), and pseudo-second-order (**c**) for the degradation of Red BF-4B dye in concentrations of 15 (■), 30 (●), 45 (▲), and 60 (▼) mg L^−1^, catalyzed by PBAT/PLA/TiO2 electrospun fibers.

After obtaining the kinetic data, the zero-order, pseudo-first-order, and pseudo-second-order models were applied to quantitatively analyze the reaction kinetics for the degradation of Red BF-4B [45]. Figure 4 shows the kinetic profiles of zero-order, pseudo-first-order, and pseudo-second-order for the degradation of the Red BF-4B dye at dye concentrations of 15, 30, 45, and 60 mg L^−1^.

From the curves of Ct, Ln (C/C_0_), and 1/C as a function of time, straight lines are obtained whose slopes correspond to the degradation rate constant (k). Table 2 shows the parameters for the models obtained at each concentration, as well as the degradation rate constants (k), the determination coefficient (R^2^), and the root mean square error (RMSE) for the studied kinetic models, which are also furnished in Table 2.

From the data presented in Table 2, it can be seen that the mathematical adjustments using the pseudo-first-order model are satisfactory for all cases, given that the experimental data at the different evaluated concentrations (15, 30, 45, and 60 mg L^−1^) fit the model better, with determination coefficient values close to 1.0 and low RMSE values.

It is also noted that after 360 min, the data of the kinetic curves are better adjusted to the linear model when compared to the beginning of the photocatalytic process. It is noticed that with each increase of 15 mg L^−1^ in the dye concentration in solution, the degradation rate decreases by an average of 23.94%. This corroborates the previously mentioned fact, since at high concentrations, it is difficult for the photons of light to reach the surface of the catalyst, and consequently, the inhibition of the formation of electron–gap pairs and generation of hydroxyl radicals occurs.

The pseudo-first-order model assumes that the dye concentration in the reaction medium is in excess, with the formation of hydroxyl radicals being the limiting factor for the reaction rate, as these radicals are responsible for degrading the organic compound [46]. Some authors have reported that the photocatalytic degradation of several organic substances, including the dye Red BF-4B, using TiO_2_ as a catalyst, follows first-order decay kinetics [47].

After the kinetic behaviors were checked, the potential for reusing the PBAT/PLA/TiO_2_ electrospun fibers and the capability of each sample of electrospun fibers were investigated during six subsequent runs, with a 600 min duration for each cycle. In terms of practical application and aiming to save the process, the catalyst’s useful life after each cycle is essential. In some situations, during its use, the catalyst can be poisoned by contaminants or its particles washed out in the water-process discarding. In their work, related to the development of a polysulfone-based report that the membrane can be easily removed from the reactor and repeatedly used in photodegradation, significantly reducing costs after treatment, unlike the use of free nanoparticles [48].

**Figure 5:**
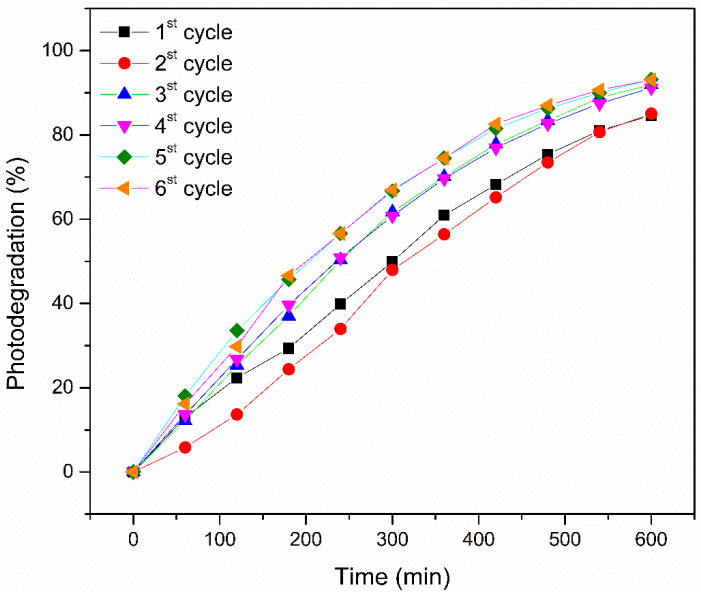
Photocatalytic activity of the PBAT/PLA/TiO_2_ polymeric film during six subsequent cycles in removing the Red BF-4B dye.

The percentage of photodegradation (or % dye removal) of the Red BF-4B dye during the cycles is shown in Figure 5.

Table 3 shows the percentage of degradation obtained for each cycle, after 600 min reaction time.

The removal efficiency of the Red BF-4B dye increased during the first three cycles, reaching 84.60%, 85.04%, and 91.94%, respectively. The fourth cycle showed removal similar to the third cycle, which was 91.18%, and finally, the last two cycles showed removal of approximately 93%. The increase in the percentage of degradation possibly occurs due to the greater exposure of TiO_2_ with the degradation cycles, because, as mentioned before, before the degradation cycles are started, the particles of the catalyst are mostly found inside the fibers.

Opposite results were found by Aoudjit [49], who studied the reuse of trifluoroethylene vinylidene fluoride (P (VDF-TrFE)) with TiO_2_ (P25) by the solvent evaporation method (solvent casting). In this study, two cycles of reuse were performed in tartrazine dye solutions, with 78% degradation of the tartrazine dye in the first and 67% degradation in the second. The authors concluded that it was impossible to carry out additional reuse, as the loss of photocatalytic efficiency occurred because the catalyst nanoparticles were not efficiently attached to the polymer matrix [49].

Another study reporting the formation of a photocatalytic filtering membrane from graphene/nylon-6 oxide using the electrospinning technique and later hydrothermal deposition of P25 nanoparticles demonstrated that the photodegradation of methylene blue, monitored for four cycles lasting 120 min, showed efficiency with a decrease of less than 10% in photocatalytic action (<10%), possibly caused by the deposition of by-product particles on the surfaces of the nanoparticles or by the washing of TiO_2_ nanoparticles from the fiber surface [50].

There are also several other papers reporting studies on the recyclability of nanocomposites that demonstrate losses in similar photocatalytic efficiencies; however, the immobilization of catalysts seems to be a great challenge to be overcome, even though there may be losses related to the large amounts of dyes that can accumulate on the surface of polymeric fibers and, consequently, decrease the active photocatalytic sites [49,51,52].

Thus, it becomes essential to verify the nanomaterial’s behavior after the photocatalysis experiments to demonstrate its full potential. Although several studies have been found on the possible detachment of photocatalytic nanomaterials from their supports, there is no knowledge of studies that investigated the properties and characteristics of these materials after the catalysis process.

**Figure 6:**
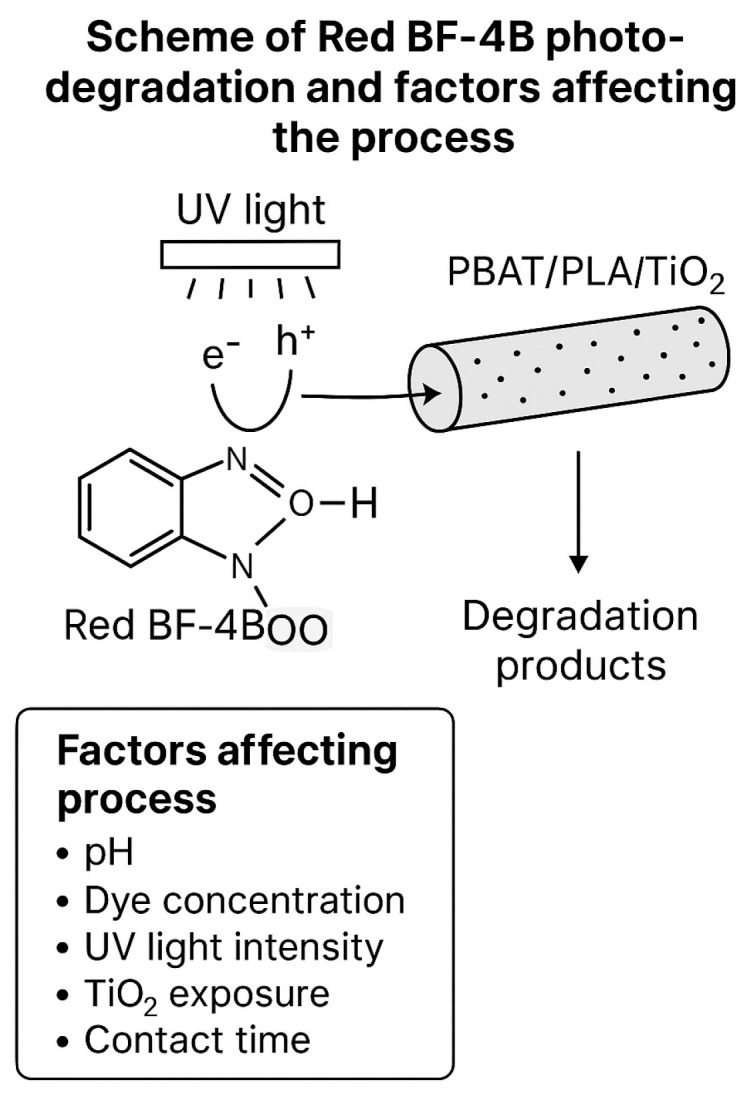
Molecular schematic of the heterogeneous photocatalytic degradation of Red BF-4B dye using PBAT/PLA/TiO_2_ electrospun fibers. Upon UV irradiation, TiO_2_ nanoparticles generate electron–hole pairs, leading to the formation of reactive oxygen species such as hydroxyl radicals (•OH). These radicals attack the dye molecules, breaking down their complex azo structures into smaller, non-toxic compounds. The efficiency of this process is influenced by factors including pH, dye concentration, UV light intensity, catalyst exposure, and reaction time.

To further clarify the photodegradation mechanism of Red BF-4B dye, a molecular schematic has been included (Figure 6). Upon UV irradiation, TiO_2_ nanoparticles embedded in the PBAT/PLA electrospun fibers become photoactivated, generating electron–hole pairs. These pairs lead to the formation of reactive oxygen species, primarily hydroxyl radicals (•OH), which attack the azo dye molecules, breaking their complex structures into smaller, non-toxic compounds. The efficiency of this process is influenced by several factors, including solution pH, dye concentration, UV light intensity, exposure of TiO_2_ due to polymer degradation, and reaction time. These factors were systematically investigated and discussed throughout the study.

**Figure 7:**
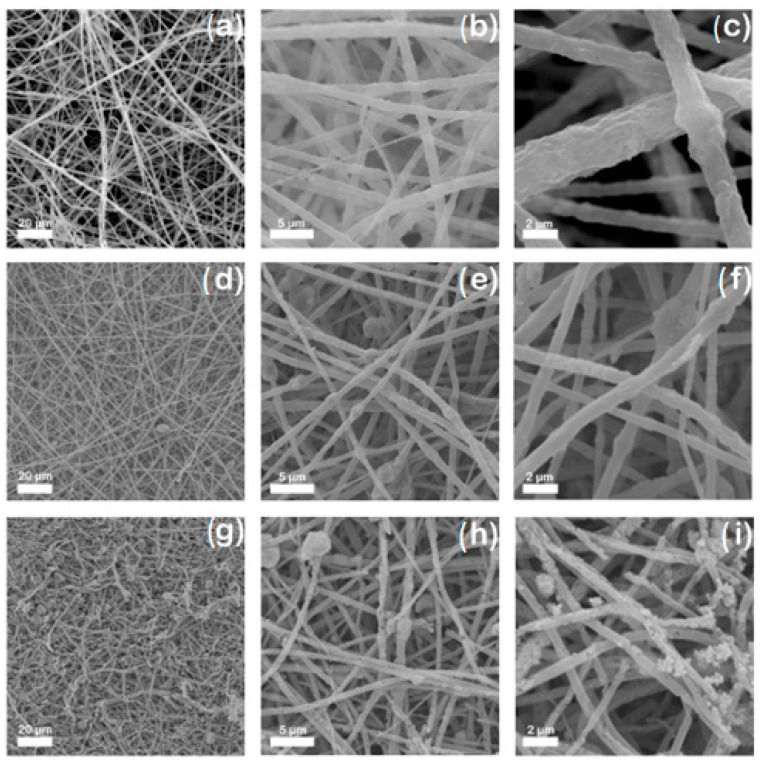
Scanning electron microscopy (SEM) images for PBAT/PLA/TiO_2_ polymeric films before photocatalytic treatment (**a**–**c**), after the 1st photocatalytic treatment cycle (**d**–**f**), and after the 6th photocatalytic treatment cycle (**g**–**i**) at magnifications of 2000×, 10,000×, and 20,000×, respectively.

After the reuse test of the PBAT/PLA/TiO_2_ electrospun fibers, morphological and surface-chemical composition characterizations were performed. The SEM images shown in Figure 7 compare PBAT/PLA electrospun fibers obtained before and after the first and sixth degradation cycles using the same sample of polymeric film.

From the comparison between PBAT/PLA/TiO_2_ electrospun fibers before the photocatalytic process and those after the 1st and 6th degradation cycles, it is observed that in both cases, the fibers continue to show cylindrical morphology. However, the roughness of the PBAT/PLA/TiO_2_ electrospun fibers appears to decrease after the 1st and 6th degradation cycles, making the TiO_2_ nanoparticles more exposed. As expected, TiO_2_ is more exposed, and the fibers present several fractures, corroborating the degradation results obtained during the reuse of the polymeric film. These results suggest that the polymer blend (PBAT/PLA) may be degrading during the photocatalytic processes.

In addition to the visual characteristics, the average diameter of the fibers was studied (Table 4). Table 4 shows a reduction in fiber diameters, mainly during the first cycle, whose decline reached 31.64% compared to PBAT/PLA/TiO_2_ fibers before the photocatalytic process. Between the first and sixth reuse cycles of the polymeric film, the reduction in diameter was smaller, 7.95%. These results reinforced the possibility of polymeric degradation.

EDS analyses were performed to better elucidate the presence and distribution of the constituents (C, O, and Ti) on the fiber’s surface after their reuse. Table 5 presents the mass percentages obtained from the EDS analysis for the studied treatments.

It is observed, through the EDS spectra, that the PBAT/PLA/TiO_2_ electrospun fibers before and after the 1st degradation cycle present similar mass percentages among the evaluated elements, with little variation. However, after the 6th cycle, a more significant reduction in carbon (C) and an increase in the percentage of titanium (Ti) on the surface of the fibers were observed. This reinforces that, during the photocatalytic process, the polymer is degrading, which exposes the Ti atoms in the polymeric fibers more, resulting in more significant degradation, as evidenced by the reuse analysis (Figure 5).

**Figure 8:**
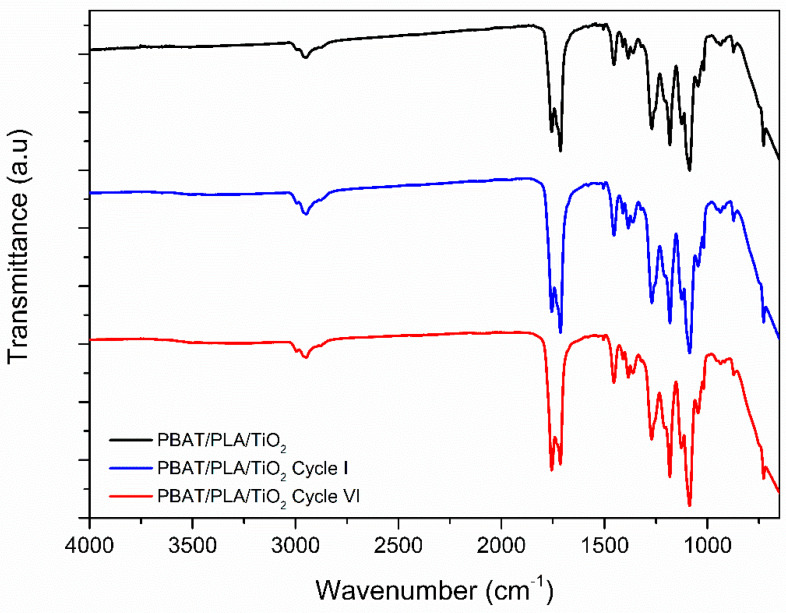
FTIR-ATR vibrational spectra of PBAT/PLA/TiO_2_ polymeric films after their use in the 1st and 6th photodegradation cycle.

To detect these changes, additional analyses, such as FTIR (Figure 8), were performed to examine potential alterations in the functional groups of the PBAT and PLA polymers.

From the FTIR-ATR vibrational spectra shown in Figure 8, it is observed that after both the 1st and 6th photodegradation cycles, the spectral bands of the functional groups of the PBAT/PLA polymers remain [27]. However, significant changes were detected in the regions of 1755 cm^−1^ and 1714 cm^−1^, which are attributed to C=O (PLA) and C–O (PBAT) stretches, respectively. The observed changes indicate that with an increase in the number of cycles, UV irradiation and the action of reactive oxygen species cause the scission of these chemical bonds, leading to conformational changes and the progressive degradation of the PBAT/PLA blend [53]. Between 1000 and 600 cm^−1^, the characteristic band attributed to the Ti-O-Ti vibration remains, confirming the stability of the semiconductor within the polymeric matrix.

As a result of the progressive degradation, the PBAT/PLA matrix becomes increasingly fragile. The increase in the first derivative (PLA) area after the sixth cycle indicates a higher rate of degradation, showing the greater fragility of the polymer chains. This accelerated degradation with an increasing number of cycles can be attributed to the continuous exposure of the polymer chains to UV radiation and to reactive species generated by photocatalysis. This is because the ongoing degradation process leads to a reduction in fiber diameter and the appearance of new fractures, which in turn exposes more of the polymer’s surface area and a greater number of embedded TiO_2_ particles to the reaction environment, creating a positive feedback loop that intensifies the material’s degradation.

**Figure 9:**
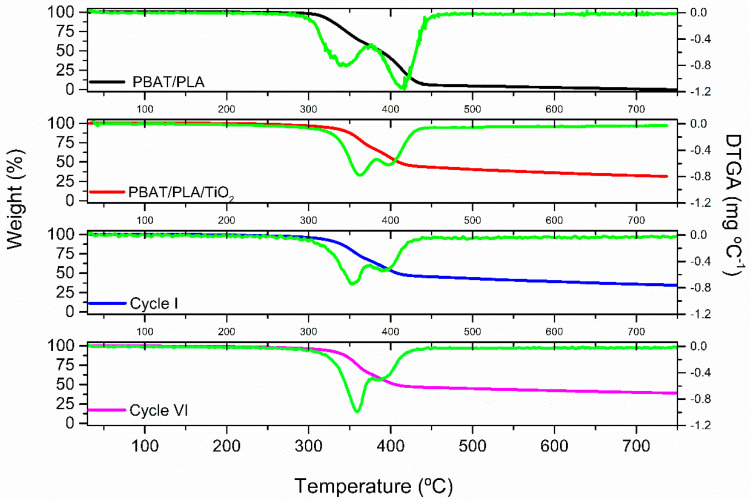
Thermogravimetric curves (TGA) and records of the derivatives of the decomposition curves (DTGA) (■) of the fibers of PBAT/PLA and PBAT/PLA/TiO_2_, before and after the 1st and 6th cycles of photodegradation.

In addition to these chemical changes, the degradation of the polymer matrix was further investigated by analyzing the thermal properties of the electrospun fibers. The functional groups’ analysis was studied through the thermal properties of the PBAT/PLA/TiO_2_ electrospun fibers. Figure 9 shows the thermograms referring to the PBAT/PLA/TiO_2_ fibers before the photocatalytic process and after the execution of the first and sixth cycles of reusing the same sample of polymeric film.

The two thermal events observed in the thermograms (Figure 9) correspond to the maximum thermal degradations of the PLA and PBAT polymers, respectively [26,54,55]. From Figure 9, it is observed that the degradation profiles changed, both due to the presence of the TiO_2_ NPs and after the reuse of the PBAT/PLA/TiO_2_ electrospun fibers in the first and sixth cycles of photocatalytic degradation. The increase in the first derivative (PLA) area after the sixth cycle indicates a higher rate of degradation, showing the greater fragility of the polymer chains. These results corroborate the conformational changes observed in the FTIR analysis (Figure 8).

Table 6 shows the maximum degradation temperatures of PLA and PBAT polymers and the percentage of degradation at 475.8 °C of PBAT/PLA and PBAT/PLA/TiO_2_ polymeric fibers before and after the 1st and 6th photodegradation cycles.

When observing the fibers’ thermal degradation residues, it is noted that the fibers containing only the polymer blend (PBAT/PLA) showed 95% degradation at 475.8 °C. When evaluating the residual mass of the PBAT/PLA/TiO_2_ fibers, it is noted that there were no changes after the studied cycles (first and sixth cycles), indicating that the presence of the TiO_2_ NPs in all composite polymeric films remains in the same amount, that is, there was no loss of TiO_2_ due to the photoreaction process.

**Figure 10:**
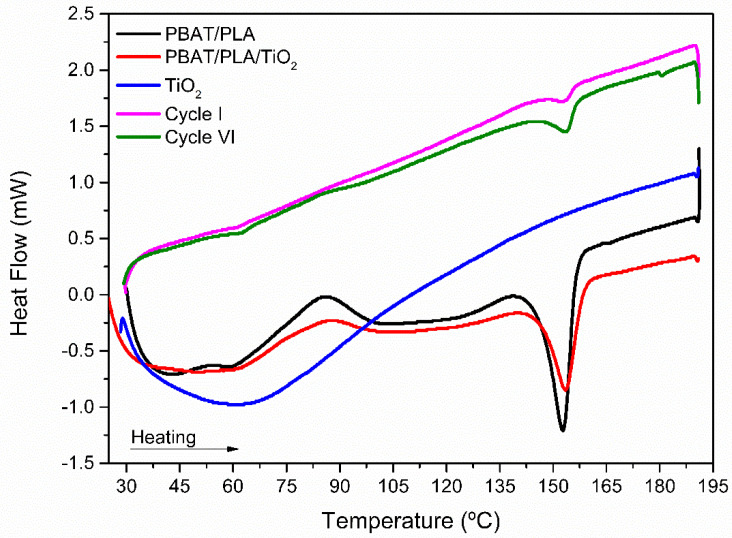
Heating thermograms (DSC) referring to the polymeric fibers of PBAT/PLA and PBAT/PLA/TiO_2_ before and after the first and sixth cycles of photocatalytic degradation of the dye Red BF-4B.

**Figure 11:**
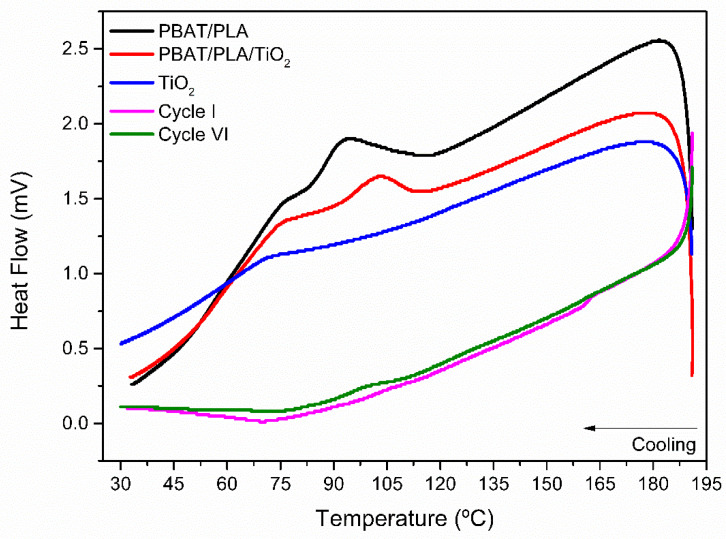
Cooling thermograms (DSC) referring to the polymeric fibers of PBAT/PLA and PBAT/PLA/TiO_2_ electrospun fibers before and after the 1st and 6th cycles of photocatalytic degradation of the dye Red BF-4B.

Figure 10 and Figure 11 show the DSC curves of the electrospun fibers with and without the incorporation of TiO_2_ NPs, and of the PBAT/PLA/TiO_2_ fibers reused after the 1st and 6th photocatalytic cycles.

In the heating thermograms (Figure 10), it is noted that the glass transition temperature of PLA (≈56 °C), in which the amorphous phase of the polymer chains acquires mobility, is still evidenced by a discrete thermal event, even after the photocatalytic treatments of one and six consecutive cycles [56,57]. In this way, a thermal event associated with the cold crystallization of PLA, at approximately 100 °C, and an event related to the fusion of the aromatic monomers in the PBAT chains, at 103 °C, both become imperceptible after exposure to photocatalytic degradation for one and six consecutive cycles. Only the thermal event at 153 °C, associated with the crystalline fusion of PLA, was observed. However, it appears with less intensity compared to before the photocatalytic processes, as can be observed (Table 7) [58,59]. The variation in the melting temperature of PLA/PBAT can be explained by a process of selective polymer degradation. Initially, during the first cycle, UV radiation and reactive species from the photocatalytic processes preferentially attack and degrade the more disorganized (amorphous) parts of the polymer. As a result, only the more perfect and stable crystalline regions remain, leading to an increase in the observed melting temperature. However, with prolonged exposure over subsequent cycles (up to the sixth cycle), the degradation process intensifies and begins to fragment even the crystalline regions, breaking them into smaller, less perfect pieces. The melting of these imperfect crystals requires less energy and occurs at a lower temperature, which explains the reduction in the melting temperature [60]. During the samples’ cooling (Figure 11), it is worth noting that the crystallization events observed in the PLA/PBAT and PLA/PBAT/TiO_2_ fibers at approximately 100 °C are no longer noticeable. These findings indicate that photocatalytic degradation causes structural/conformational changes in the polymers, leading to the weakening of the polymer fibers, as also observed in previous analyses and discussed earlier.

**Figure 12:**
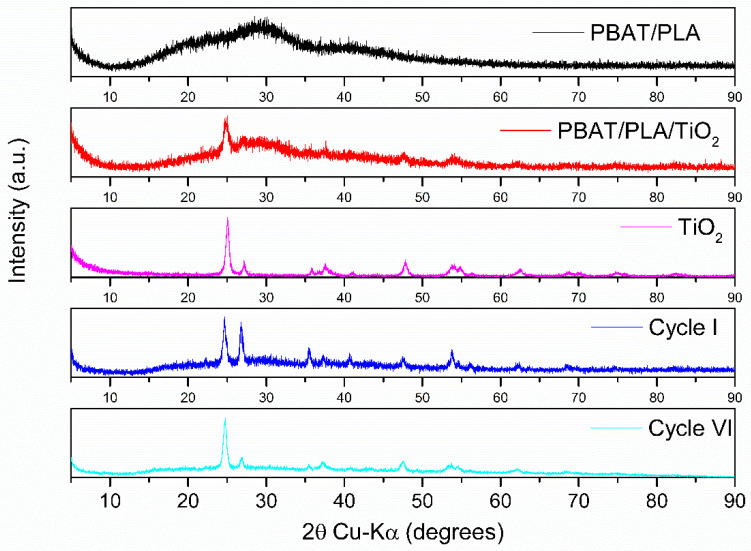
X-ray diffractograms of TiO_2_ NPs, polymeric fibers of PBAT/PLA, and PBAT/PLA/TiO_2_ before and after the 1st and 6th cycles of photodegradation of the Red BF-4B dye.

XRD analyses (Figure 12) were conducted to assess the crystallization of PBAT/PLA/TiO_2_ fibers after reuse.

The X-ray diffractograms (Figure 12) demonstrate that after the photocatalytic cycles (first and sixth cycles), the TiO_2_ NPs are more exposed, indicating a probable loss of polymeric material during this process. Additionally, this greater exposure corroborates the more significant degradation shown in Figure 5 during the reuse cycles.

The X-ray diffraction (XRD) patterns of the PBAT/PLA/TiO_2_ electrospun fibers revealed characteristic peaks corresponding to the anatase and rutile phases of TiO_2_. The anatase phase was identified by reflections at 2θ ≈ 25.3°, 37.8°, 48.0°, and 55.1°, which correspond to the (101), (004), (200), and (211) planes, respectively. The rutile phase was confirmed by peaks at 2θ ≈ 27.4°, 36.1°, and 54.3°, associated with the (110), (101), and (211) planes. After the 1st and 6th photocatalytic cycles, the intensity of these peaks increased, indicating greater exposure of TiO_2_ due to polymer degradation. Additionally, the broad halo observed around 2θ ≈ 16–22° is attributed to the amorphous regions of the PBAT/PLA polymer blend. The reduction in this halo after repeated cycles suggests a loss of polymeric material and a shift toward increased crystallinity of the exposed TiO_2_ phase. These findings support the morphological and thermal analyses, confirming the structural evolution of the material during reuse.

The characterization results obtained after the degradations of one and six consecutive cycles show that there is possible degradation of polymeric material, demonstrating that the TiO_2_ NPs remained immobilized in the fibrous structures of the PBAT/PLA matrix even after reuse for six cycles, presenting excellent performance without loss of the semiconductor. In addition, it is essential to note that the polymer blend used in this research is considered a bioplastic, bio-based, composed of 45% by weight of poly (lactic acid) (PLA) and 55% by weight of poly (butylene adipate-co-terephthalate) (PBAT), with characteristics of biodegradability and compostability [61,62].

The crystallinity of the PBAT/PLA/TiO_2_ electrospun fibers was evaluated through X-ray diffraction (XRD) and differential scanning calorimetry (DSC). The XRD patterns revealed distinct diffraction peaks corresponding to the anatase and rutile phases of TiO_2_, as well as broad halos indicative of the semi-crystalline nature of the polymeric matrix. After the first and sixth photocatalytic cycles, an increase in the intensity of TiO_2_ peaks was observed, suggesting a progressive degradation of the polymer and greater exposure of the embedded nanoparticles. This is consistent with the DSC results, which showed a reduction in the melting enthalpy of PLA from 7.58 J/g (before use) to 0.61 J/g and 1.51 J/g after the first and sixth cycles, respectively. These changes indicate a decrease in the degree of crystallinity, likely due to chain scission and conformational rearrangements induced by UV irradiation and reactive oxygen species during photocatalysis. The loss of crystallinity contributes to the observed morphological fragility and supports the hypothesis of polymer degradation over successive reuse cycles.

Although morphological changes such as fiber thinning and surface fracturing were observed after the 1st and 6th photocatalytic cycles, no significant mass loss was detected. Thermogravimetric analysis (TGA) confirmed that the residual mass at 475.8 °C remained stable, indicating that the TiO_2_ nanoparticles were retained within the polymeric matrix. This suggests that the degradation primarily affected the surface structure without compromising the overall mass of the membrane. Consequently, mass change data were not presented, as the structural integrity and catalytic performance of the fibers were preserved throughout the reuse cycles.

Therefore, using the proposed material for remediating textile effluents containing dyes would prevent the emission of plastic molecules and synthetic microplastics, which require long periods to decompose, into the environment [61]. In contrast, biodegradable bioplastics undergo significant microbial biodegradation within a short period under favorable conditions, breaking down into water, biomass, methane, and/or carbon dioxide [63].

The photocatalytic performance of the PBAT/PLA/TiO_2_ electrospun fibers developed in this study was compared with other TiO_2_-based systems reported in the literature. For instance, electrospun TiO_2_ nanofibers modified with graphitic carbon nitride sheets achieved enhanced degradation of organic pollutants under solar light, but required complex doping strategies and showed limited recyclability [64]. Similarly, Ag_2_S/TiO_2_ composite fibers reached up to 70.54% degradation of tetracycline under visible light, with performance declining after five cycles [65]. In contrast, our PBAT/PLA/TiO_2_ fibers demonstrated degradation efficiencies exceeding 80% for Red BF-4B dye and maintained high performance over six reuse cycles (up to 93.22%), without significant loss of TiO_2_ or structural integrity. Moreover, the use of biodegradable and compostable polymers (PBAT/PLA) offers an environmentally friendly alternative to petroleum-based supports, reducing the risk of secondary pollution. These results position our material as a competitive and sustainable option for wastewater remediation applications.

## 4. Conclusions

The application of PBAT/PLA/TiO_2_ fibers in catalytic tests for the removal of Red BF-4B dye is promising and highlights the specific characteristics of this process. The kinetic profiles indicate that the highest efficiencies are achieved at lower dye concentrations, and the pseudo-first-order model fits the experimental data satisfactorily. The electrospun PBAT/PLA/TiO_2_ fibers can also be reused, demonstrating their viability and effectiveness in dye removal. Although changes in the fibers’ properties indicate degradation of the polymeric material, the fibers developed in this study stand out as excellent alternatives. They exhibit a high dye removal rate, are biodegradable and compostable, and allow for the reuse of the semiconductor (TiO_2_ NPs). These features demonstrate the potential of the fibers to address environmental challenges.

Thus, the electrospun polymeric film based on PBAT/PLA with TiO_2_ nanoparticles is a promising material for the photodegradation of Red BF-4B dye, achieving degradation rates above 93%. Moreover, it can be reused for at least six cycles without loss of TiO_2_ nanoparticles to the reaction medium, which is highly significant as it minimizes additional filtration and treatment steps required to remove these particles.

It is important to emphasize that the use of biodegradable and compostable polymers (PBAT/PLA) offers an environmentally responsible alternative to conventional supports, minimizing the risk of secondary pollution and contributing to sustainable wastewater treatment technologies. Compared to other TiO_2_-based systems, the developed fibers stand out for their simplicity, efficiency, and durability, positioning them as a promising solution for textile effluent remediation and broader environmental applications.

## Figures and Tables

**Table 1:** The pH values of the Red BF-4B dye solution before and after the photocatalytic process with PBAT/PLA/TiO_2_ electrospun fibers.

pH_initial_	pH_final_
4.00	3.67
6.00	4.88
8.00	6.33
10.00	7.02

**Table 2:** Kinetic parameters obtained from the linearization of the zero-, first-, and second-order decay models for the degradation of the dye Red 4B-BF.

Kinetic Model		15 mg L^−1^	30 mg L^−1^	45 mg L^−1^	60 mg L^−1^
Zero-order	Linear equation	$y=-2.2510^{-2}x+14.86$	$y=-4.1210^{-2}x+29.05\;$	$y=-5.8210^{-2}x+46.84\;$	$y=-6.4810^{-2}x+62.34$
	Rate constant k (min^−1^)	$2.25\times 10^{-2}\;$	$4.12\times 10^{-2}\;$	$5.82\times 10^{-2}\;$	$6.85\times 10^{-2}$
	R^2^	0.99	0.99	1.00	0.99
	RMSE	0.36	0.73	0.56	1.67
Pseudo-first-order	Linear equation	$y=3.5410^{-3}x-0.34\;$	$y=2.9210^{-3}x-0.18\;$	$y=2.3110^{-3}x-0.21\;$	$y=1.8610^{-3}x-0.17$
	Rate constant k (min^−1^)	$3.54\times 10^{-3}\;$	$2.92\times 10^{-3}\;$	$2.31\times 10^{-3}\;$	$1.86\times 10^{-3}$
	R^2^	0.97	0.99	0.97	0.96
	RMSE	0.11	0.06	0.06	0.06
Pseudo-second-order	Linear equation	$y=6.9810^{-2}x-3.52\times 10^{-2}\;$	$y=2.4010^{-4}x+3.70\times 10^{-3}\;$	$y=1.0010^{-4}x+9.01\times 10^{-3}\;$	$y=5.3710^{-5}x+1.03\times 10^{-2}$
	Rate constant k (min^−1^)	$7.00\times 10^{-4}\;$	$2.40\times 10^{-4}\;$	$1.00\times 10^{-4}\;$	$5.00\times 10^{-5}$
	R^2^	0.80	0.90	0.90	0.88
	RMSE	0.06	0.01	0.06	0.00

**Table 3:** Percentage of degradation obtained in six consecutive cycles of photodegradation of Red BF-4B dye with PBAT/PLA/TiO_2_ fibers.

Cycle	Photodegradation Percentage
1st	84.60
2nd	85.04
3rd	91.94
4th	91.18
5th	93.22
6th	93.07

**Table 4:** Average diameters of the PBAT/PLA/TiO_2_ fibers and after their use in the 1st and 6th cycles of photodegradation.

	PBAT/PLA/TiO_2_ Before Use	PBAT/PLA/TiO_2_ After 1st Cycle	PBAT/PLA/TiO_2_ After 6th Cycle
Average diameter	1.12	0.77	0.71
Standard deviation	0.26	0.22	0.14

**Table 5:** Mass percentage of carbon (C), oxygen (O), and titanium (Ti) indicated by the EDS technique in the polymeric fibers of PBAT/PLA/TiO_2_ before and after the 1st and 6th degradation cycles.

Samples	C (%)	O (%)	Ti (%)	Total (%)
PBAT/PLA/TiO_2_	31.99 ± 0.97	40.07 ± 1.06	27.94 ± 1.51	100.00
After 1st cycle	38.04 ± 0.87	33.20 ± 1.18	28.76 ± 0.32	100.00
After 6th cycle	23.38 ± 1.58	33.68 ± 2.90	42.94 ± 4.49	100.00

**Table 6:** Maximum degradation temperatures of PBAT/PLA and PBAT/PLA/TiO_2_ electrospun fibers before and after the first and sixth cycles of photocatalytic reuse of the polymeric film.

Samples	Tm PLA (°C)	Tm PBAT (°C)	% Degradation T_475_._8 °C_
PBAT/PLA	345.0	413.3	95.0
PBAT/PLA/TiO_2_	362.5	397.1	41.8
1st cycle	351.4	389.1	44.3
6th cycle	359.1	386.4	45.5

**Table 7:** Melting temperature values of the PLA polymer and their respective heats for the PBAT/PLA and PBAT/PLA/TiO_2_ fibers and the 1st and 6th photodegradation cycles.

Sample	T_Melting_ (°C)	Heat (J/g)
PBAT/PLA	152.5	12.76
PBAT/PLA/TiO_2_	153.9	7.58
1°	159.0	0.61
6°	153.6	1.51

## Data Availability

The original contributions presented in this study are included in the article. Further inquiries can be directed to the corresponding author.
